# What challenges do people face in response to the long-term use of face masks?

**DOI:** 10.5339/qmj.2022.fqac.13

**Published:** 2022-03-28

**Authors:** Yosra Raziani, Ahmad Nazari, Sheno Raziani

**Affiliations:** ^1^Department of Nursing, Komar University of Science and Technology, Sulaymaniyah, Kurdistan Region, Iraq E-mail: yosra.anvar@komar.edu.iq; ^2^Department of nursing, Sanandaj branch, Islamic Azad University, Sanandaj, Iran; ^3^Master of Psychology, Department of Literature and Humanities, Graduate of Malayer University, Malayer, Iran

**Keywords:** challenges, face masks, long-term use

## Abstract

Background: The world is ceaselessly adapting to the ever-changing circumstances surrounding the COVID-19 pandemic, and face masks have become a part of our daily routine. Considering the fact that face masks are here to stay, there are challenges people experience regarding wearing them. In this descriptive study, we aimed to investigate the frequent outcomes of long-term use of face masks in the general population.

Methods: Twenty-five nursing students who attended the university wearing face masks owing to the current pandemic were selected. The data were collected using semi-structured interviews. The interviews were transcribed verbatim and analyzed according to the qualitative content analysis according to the Graneheim and Lundman method.

Results: The mean current age of the students was 20 (standard deviation [SD] = 2.5) years, and 60% of the students were women. The two main themes that emerged during data analysis were:

1. Physical health related problems, which included 3 categories; a) skin reactions, b) respiratory consequences, and c) visual complaints

2. Socioeconomic problems, including the 3 categories of; a) communication failure, b) personal style and appearance, and c) economic factors.

Conclusion: We support the necessity of face masks for a safe reopening of communities. Considering their advantages, some basic measurements are necessary to reduce the abovementioned concerns.

